# A digital advocate? Reactions of rural people who experience homelessness to the idea of recording clinical encounters

**DOI:** 10.1111/hex.12492

**Published:** 2016-09-07

**Authors:** Stuart W. Grande, Mary Ganger Castaldo, Elizabeth Carpenter‐Song, Ida Griesemer, Glyn Elwyn

**Affiliations:** ^1^ The Preference Laboratory The Dartmouth Institute for Health Policy and Clinical Practice Lebanon NH USA; ^2^ The Dartmouth Psychiatric Research Center Lebanon NH USA

**Keywords:** patient‐provider communication, recording clinical encounters, shared decision‐making

## Abstract

**Background:**

Are the benefits of recording clinical encounters shared across different groups, or do they vary based on social position? Studies show that educated patients record their clinical visits to enhance their experience, but very little is known about recording benefits among “hard‐to‐reach” populations.

**Objective:**

To examine the reactions of homeless people to the idea of using a smartphone to record their own clinical encounter, either covertly or with permission from their physician.

**Method:**

We conducted semi‐structured interviews with individuals at a temporary housing shelter in Northern New England. A thematic analysis identified themes that were iteratively refined into representative groups.

**Results:**

Eighteen (18) interviews were conducted, 12 with women and six with men. Initial reactions to clinical recordings were positive (11 of 18). A majority (17 of 18) were willing to use recordings in future visits. A thematic analysis characterized data in two ways: (i) by providing reliable evidence for review, they functioned as an advocacy measure for patients; (ii) by promoting transparency and levelling social distance, this technology modified clinical relationships.

**Discussion:**

Recordings permitted the sharing of data with others, providing tangible proof of behaviour and refuting misconceptions. Asking permission to record appeared to modify relationships and level perceived social distance with clinicians.

**Conclusions:**

We found that while many rural, disadvantaged individuals felt marginalized by the wide social distance between themselves and their clinicians, recording technology may serve as an advocate by holding both patients and doctors accountable and by permitting the burden of clinical proof to be shared.

## 1 Introduction

Are the benefits of recording clinical encounters shared across different groups, or do they vary based on social position? It is already clear that smartphones are being used by some patients to record clinical encounters,^1^, ^2^ particularly by those empowered to do so.^3^ An exploration of why patients record suggests the majority seek to enhance the value of their encounters, to relisten and to share the recordings with others. This seems particularly true of encounters where there is complex information to consider, such as in changing medication or following new advice about treatment.^4^

Few studies have examined the extent to which patients are recording clinical encounters,^5^, ^6^ yet there are indications that recording might be occurring more often than previously thought.^2^, ^7^ Could it be that the willingness to record, to anticipate the potential benefits, is predicated on social position? In an earlier survey, more education was associated with more willingness to request permission to record overtly, and covert recording was more likely to be undertaken where there was more distance between the social position of the patient and the professional.^2^

Qualitative work has found that patients from educated and wealthy strata hesitate to ask questions and disagree with medical professionals.^8^ It is likely that this hesitancy is even greater as social distance increases.^9^ Might it also be possible that the use of technology to empower patients—patients using smartphones to record—is also affected by social distance? Or might the use of a technology to record a clinical encounter—given that it may not require as much interactional assertiveness—leads to patients feeling more in control?

Tools that have been advocated for use at the point of care to improve patient–clinician interactions may help to reduce social distance.^10^ Yet, few studies have explored their benefits among “hard‐to‐reach” populations.^11^ We know that educated patients have been using technologies like smartphones to record clinical encounters, sometimes covertly.^1^ Rationale for this among activated patients appears based on a lack of trust and previously negative experiences, as well as a fear of being denied permission to record and/or losing access to care as a result of asking to record.^2^

According to Good,^12^ fervent belief in technology mimics what she calls the “medical imaginary,” the idea that medical innovation offers solutions, when real change is often onerous and time‐consuming. In the light of Good's concepts of the “medical imaginary,” we wanted to explore reactions to the idea of using smartphones to record clinical encounters among the most disenfranchised—those who have experienced housing insecurity. While some patients benefit from recording their clinical encounters, we could not find any studies that have explored this issue among the most disadvantaged. We were interested in understanding the potential for recording clinical encounters as a patient empowerment strategy among economically vulnerable, rural populations.

The aim of this study was to examine the reactions of homeless people to the potential of using a smartphone to record their clinical encounter, either covertly or with permission.

## 2 Methods

### 2.1 Setting

We conducted a qualitative, semi‐structured interview study with residents of a shelter for those who need temporary housing because of homelessness, located in New England, USA. The shelter serves over 10 000 people a year and provides temporary shelter, programming, food and clothes for families and adults in need. It has the capacity to house eight families, and typical stays for families are 2–3 months. A majority of guests leave the shelter able to find and maintain permanent housing.

### 2.2 Method and data collection

**Figure 1:**
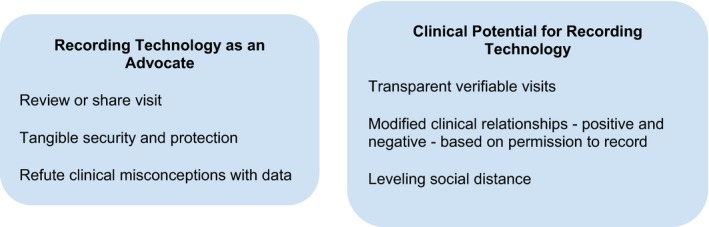
Advocacy model of clinical recording technology

Because we wanted to explore detailed views about a pre‐specified topic, we elected to use semi‐structured interviews (Fig. 1). Qualitative interviews 13 “foster learning about individual experiences and perspectives on a given set of issues” (p. 314), and we were interested in how the residents would react to the idea of using smartphones to record encounters. Patton^14^ states this method “…begins with the assumption that the perspective of others is meaningful, knowable, and able to be made explicit” (p. 278). We therefore designed our interview schedule to be neutral about the topic (see Appendix). This enabled us to characterize reactions to the idea of recording clinical encounters and to explore what, if any, support participants might need before they consider using recording technologies in clinical visits with their doctor. We also asked participants about harms and benefits of recording, if they would be willing to record covertly and if it is something they had ever consider doing. Further, we asked how a recording might be useful and whether they would be willing to try recording a clinical visit in the future. Interviews were recorded and transcribed.

### 2.3 Recruitment and data collection

We wanted to talk to individuals who had experienced either an acute housing crisis or long‐term situational poverty. Previous work with homeless populations suggested that individuals in these situations have limited access to health care and poor continuity of care, experience fragmented and uncoordinated services and used emergency departments for primary care. We planned to interview 30 such individuals. We received institutional review board approval from The Dartmouth Committee for the Protection of Human Subjects (CPHS) to conduct this research.

After receiving ethical approval, we recruited shelter residents with volunteer shelter staff. Participants were eligible if they were 18 years or older and lived at the shelter. The research team gave study information to potential participants, who read or were told about the study and were offered time for questions. Residents interested in participating were consented by the research team (SG, MC and IG), interviewed and recorded. Following the interview, participants received a $10 gift card.

### 2.4 Data analysis

We conducted a thematic analysis of interviews.^15^ Interview transcripts were read, qualitatively coded, reviewed and labelled.^16^ Qualitative coding^17^ “mean[t] naming segments of data with a label that simultaneously categorizes, summarizes, and accounts for each piece of data” (p. 43). The initial stages of coding involved a process of labelling portions of text to identify and formulate ideas, themes, and issues.^18^ Coding is the “pivotal link” between data collection and interpreting the meaning of qualitative data.^17^ Through on‐going immersion in the data set, members of the team (SG, MC, ECS) advanced to “focused coding,” which utilized the prominent themes as the basis for more fine‐grained analyses.^18^ Throughout discussion and consensus, broad conceptual codes were gradually refined to ensure that our analytic categories were credible and “trustworthy”.^19^

## 3 Results

Our initial goal was to interview 30 participants. As we began the iterative process of analysing data, we felt that we had reached saturation by participant 15, uncovering no new information or nuance in individual accounts. However, potentially due to our recruitment strategy, waiting in common areas and talking with potential participants over coffee, there was strong interest in participating, sharing points of view and reacting to the importance of technology. We noticed quickly that most potential participants approached our research team out of curiosity. Many conversations began in the common areas, suggesting that the topic, using technology to improve communication with the health care system, was salient and meaningful. Recruiting in the common area, meeting people face to face over coffee, challenged notions of social distance. It also proved to a superior recruitment method compared to initial efforts that relied solely on posters, fliers and shelter staff.

Eighteen interviews were conducted, lasting between 15 and 30 minutes. Twelve were women and 11 were between 36 and 60 years of age (Table 1). Many (11 of 18) had positive initial reactions to use of recordings; less than half (7 of 18) were negative or conditional. A majority (12 of 18) felt they would record in the future only if the recording was overt (10/12), not covert. A smaller group (5 of 18) felt they would only record if certain conditions were met, while only one participant remained opposed (Table 2).

**Table 1:** Demographic profile of interviewed participants (n=18)

	Number
Gender	
M	6
F	12
Age	
18–25	1
26–35	6
36–45	5
46–55	3
>56	3
Race/Ethnicity	
White/Native American—Alaskan Native	2
Black/Non‐Hispanic	2
White/Non‐Hispanic	13
Other/Hispanic	1

**Table 2:** Comparison of initial and informed beliefs to recording

	Number
What is your initial reaction to recording a doctor's visit?	
Positive	11
Negative	3
Conditional	4
Would you record your doctor's visit in the future?	
Yes, I would	12
Overtly	10
Covertly	2
I might	5
I would not	1

A thematic analysis (Fig. 1) of interview transcripts characterized clinical recordings in two ways: (i) by providing reliable evidence for review, they functioned as an advocacy measure for patients; (ii) by promoting transparency and levelling social distance, this technology modified clinical relationships.

### 3.1 Recording technology as an advocate

The interviews revealed a palpable social distance between participants and their doctors. Given the nature and context in which many of the participants lived—often noting financial insecurity, cost fears and rampant mistrust—recording technology was seen as a means to prove they deserve to be taken seriously. One participant commented *“if you can't have an expert with you, the tape recorder is excellent,”* (ID15). The notion of verifying the clinical visit with a recording was seen as *“a smart idea,”* and if done openly, may change how medical professionals perceive them.

I know from my own personal experiences, things have been twisted or overlooked… it's their word against yours. And, them being a doctor, who they going to believe? Some low income [person]?

(ID11)

### 3.2 Review or share visit

The most salient reaction to recording was using it to review and rethink the visit. The desire to use recordings to certify unmet need offered a new opportunity—having control over an aspect of their life normally out of their control.

I went up there [hospital], sat in an emergency room for I don't know how many hours. What I got was Doogie Howser. That boy was an intern. I am not looking for advice, I was going for rehab.

(ID6)

He further explained that a recording might provide some modicum of control,

either you have evidence that what you expected did not go on, or the doctor has evidence that he was perfectly exactly within the line of where he's supposed to be.

(ID6)

One participant explained a struggle to recall information. For him, recording was a *“security guide for memory”* (ID2). When asked if she might listen again to a recording another participant said,

I think so, because, it would make me understand more about that, and … if I had a recording to tell me more about my disease, maybe my mind would think, no don't do it.

(ID4)

When participants considered the opportunity recordings afford to share and get feedback, the benefits of relistening emerged.

Did I hear this person correctly? Let me bring it to a lawyer. Let me let my friend hear it. Are they hearing the same thing that I'm hearing?

(ID9)

Not only was a recording seen as useful for memory, there was also a recognition that listening to yourself (either the doctor or patient) could disrupt normative thinking enough to modify behaviour.

### 3.3 Tangible security and protection

Many participants felt that it was inappropriate for medical professionals to say one thing and do another. Most felt undervalued and had an acute preoccupation with ethics—determining right from wrong. Several participants recalled incidents in which they perceived clinicians to have either deliberately or negligently breached a promise (failing to listen to a patient's concerns). This reinforced feelings of vulnerability. In the presence of a recording device, participants felt more secure: *“It made me feel more open like I have back up now”* (ID18).

Some felt that a recording could be useful for protection against a clinician who might do or say something inappropriate.

Well I have this. Not blackmail, obviously, but I do have this and I can go higher up and show them this is how inappropriate you're being but first let them know there's an issue

(ID13)

The idea that a recording would be used to “catch” a clinician in a mistake was less important than the need to take care of one's self. “*I'm protecting my own hide. I'm looking out for myself”* (ID16). The recording could also be used to protect participants from miscommunication or perceived abuse. A majority suggested that recordings had security benefits for both patients and clinicians.

I think it would benefit both parties greatly… If there's a recording of how you acted, there's a defense or not.

(ID6)

### 3.4 Refute clinical misconceptions with data

Across interviews, reactions to recordings were positive. Telling the truth was paramount as many saw recordings as one way to prove their honesty. Participants shared narratives shaped by coarse interpersonal communication. Under a scaled veneer of misfortune, many spoke to the most basic element of human communication—trust. An open recording, for example, was seen as having the potential to transcend assumptions,

I think it's a good thing because it makes you think that he's more professional… he's not just talking out of his mouth, and he is taking you seriously…

(ID5)

Many felt that most doctors do not trust them—perhaps were even suspicious. Under these circumstances, a recording might prove what another clinician had said,

The doctor may be more apt to agree with other doctors’ opinions if they're being recorded and you're stating and have written documented proof that this doctor is stating that.

(ID7)

Trust in a clinical relationship was considered implicit unless it had previously been broken. When asked if she would record again, after a recent breach of trust, one participant said of her doctor, *“Oh yeah, I don't trust that woman”* (ID8).

A desire to authenticate previous experience was common. If the clinical relationship was stressed or individuals felt vulnerable, then recording was seen as evidence. When asked whether she would be willing or not to record, one participant said,

I would like to go do that, because as a black girl, and as a girl living in a homeless shelter, I know the treatment I would be getting would probably be very different from what it could be if it was in the other case

(ID2)

The recording provided her with a sense of protection, if she were to feel vulnerable again.

Participants stressed the need for evidence to verify that what they said was true. Perceptions that physicians attributed homelessness to personal failures compelled a need to explain themselves, *“I wish I could tape myself when I wake up screaming, just to let the doctors know how bad it is”* (ID17).

### 3.5 Clinical potential of recording technologies

We found that questions about the benefits of covert vs open recording elicited strong negative responses. While participants thought that a recorded doctor visit could be both pragmatic and personal, the idea of trying to record without permission seemed to challenge a deep seated moral principle that lying to your doctor was wrong. *“I definitely would not hide it”* (ID1) and another said, *“…my conscience won't allow it”* (ID15). Unless recordings were conducted in the open, they would undermine the clinical relationship. Despite some hesitation, many cited the potential that recordings done in the open could mutually benefit both the patient and the doctor.

… it's a good thing because it makes you think that he's more professional, he's not just talking out of his mouth, and he is taking you seriously. You also know that he's got that record, he can go back, which is why he does it, in case he does forget something

(ID5)

### 3.6 Transparent verifiable visits

Having a record of a conversation was seen to assure transparency in the encounter, as one participant said, *“Without the device it's a he said, she said”* (ID9). A key advantage of open recording was seen in the potential to change the clinical conversation. By making the act of recording public through asking and receiving permission, the participants speculated that they would elicit better quality care. As one participant indicated, if she had only recorded her doctor's they *“…would have been on their toes, and they would have actually done the right thing”* (ID2). In contrast, commenting on the futility of covert recording one participant noted,

I don't know if that's even legal but if it is and you're recording it, obviously the doctor doesn't know. It's not going to change anything he's going to do

(ID7)

The covert use of recordings was widely rejected as being “sneaky,” *“I firmly believe in honesty, straight forward. It's better to get an honest kick in the butt than a sweet lie”* (ID11). Authenticity and direct communication appeared to matter most.

I don't go for the business of sliding something in my pocket book or in my pocket and recording because that's being sneaky… like you're trying to entrap them.

(ID15)

When asked to clarify differences between overt and covert recording, one participant commented on capturing the “whole truth” as opposed to one constructed in full view. *“If you're recording it privately you might hear the whole truth of everything, how these people are very dismissive, is that the word?”* (ID2). However, given the option, recording openly was preferred.

### 3.7 Modified clinical relationships—positive and negative—based on permission to record

Some believed that routine clinical recording could improve the clinical experience.

If every single conversation between doctors and patients were recorded and then a certain amount of those were able to be released, it's going to improve the doctor‐patient … experience

(ID12)

Communication as equals was recognized as a means to declare themselves active participants in the medical encounter. Putting the intention to record on the table, both literally and figuratively, acts to establish readiness to engage as partners.

If you let them know this is what I want to do out in the open. If the doctor's not fine with it too bad. This is what I want to do so I can understand later because I don't understand what you're saying.

(ID1)

Participants acknowledged clinician's expertise and displayed sensitivity to treating health providers with respect. Many recognized that recording a doctor's visit has two main concerns. (i) Depending on use, some felt it could, *“…either invade on your privacy, or it can benefit you because of something that went on there and it was recorded”* (ID6). (ii) Others believed that recording might divide patients and clinicians. One participant worried it could potential breach doctor–patient confidentiality.

You can't breach your own confidentiality but if you bring this to somebody else and let them listen to it, now more than just your doctor and patient know

(ID7)

When asked by the interviewer, “How do you think they would react if you brought in or I brought in a recording device?” one participant answered, *“I think they would be insulted.”* (ID9). Most participants agreed it would come down to how they asked their doctor about recording.

I guess it all depends on your… how you present it, your whole character with it. It could either be taken as you were trying to trap me with something, or they just need to go back and reference to what we had talked about

(ID11)

### 3.8 Levelling social distance

Many of those we interviewed shared their discomfort with the social distance between themselves and their doctors. Reactions had tinges of defensiveness, *“…you know how doctors have their school papers all on the wall? You couldn't even see the wall because it was covered,”* (ID1) suggesting previous negative experiences contributed to feeling undervalued or ignored. Many had concerns of feeling “stupid” or not being as educated as the doctor and needing to have the conversation “dumbed down.” Insecurity may add to the social distance between a participant and their doctor,

A lot of times I have a hard times with the words…to describe what I want to say, especially to doctors because they use those big words that I can't even repeat.

(ID3)

When we asked, “How would they know if their doctor respected them as a patient?” the general response was *“…if he didn't hurry through the exam…I guess not using a lot of scientific jargon…just the manner, if he acts good natured…”* (ID5). Given the potential of open recording to promote more transparency, participants supported the use of a recording device for more authentic communication.

## 4 Discussion

We found that recording technology may not only serve as an advocate for the most vulnerable, it also has the potential to improve clinical practice. A majority believed if recording was done openly, it could be used to review or share clinical experience, overcome clinical misconceptions and be seen as good, reduce social distance and ultimately improve their relationship with their doctor. Unfortunately, many rural disadvantaged are further marginalized by a significant social distance between themselves and their clinicians, which creates a dynamic where many individuals feel insecure, needing to “prove” their worth. In our sample, a majority believed recording openly or with the clinician's permission was necessary for achieving potential benefits and expressed deep concern that if they hid recording activity from doctors, they risked being seen as “sneaky,” further contributing to insecurity and mistrust.

Being perceived as deficient provoked a lack of confidence and perpetuated misunderstandings about social standing. As one participant said, they would be more inclined to record a visit if they felt they were being ignored. It was not a need to catch the doctor at fault, but rather to prove their value and experience. *“I would do it if I was being done wrong and I've been reaching out for help and no one is believing me or understanding me then I would do it”* (ID 18). The value of asking permission to record seems indicative of past experiences of feeling undervalued or under suspicion. While some participants may have recognized the potential of recordings to level social distance, many were concerned about gaining permission to record as a way to prove their good intentions. Most shared an aspiration to be seen as both worthy of trust and honest.

The participants in this sample live difficult lives. They are often seen as outsiders to the general population and feel very much on the periphery.^7^ Consequently, recording was not recognized as an empowerment strategy. The need to validate negative and often hidden experiences by placing a recording device on the table was viewed as an opportunity to disrupt clinical assumptions. By asking permission and being clear about motivations, participants imagined recording as a means to build trust and improve clinical relationships.

There were strengths and weaknesses to our method. We collected interviews over five months with four different researchers, which likely reduced fidelity and reliability of the interview guide. Yet, the use of the skills and expertise of different interviewers also added depth to conversations and lent perspective to the analysis of data. Audio recordings were transcribed by a third party service and consequently limited the preliminary analysis of data. However, as the team who conducted the interviews also performed the main analysis, this provided sufficient immersion in the data. By recruiting participants from only one homeless shelter in New England, our sample was predominantly White and Non‐Hispanic. At the same time, we believe the underlying features within the findings of being unheard, desire for validation and proving worth are reflective of marginalized groups.

## 5 Conclusions

While recent attention has focused on use of recordings in clinical encounters to improve patient empowerment, we believe this is the first study to investigate this same question among those facing grinding poverty and housing insecurity in a rural area. Previous work found that among moderately educated and financially secure individuals, recordings enhanced the clinical encounter and added to feelings of empowerment and interest in reviewing and sharing recording with others.^2^ Our findings support a portion of these findings, recognizing there are meaningful differences between social groups. The most striking difference is the perceived value of covertly recording encounters. In the sample from the UK, covert recording was seen as necessary to counter previously negative experiences, suggesting empowered patients may feel they are owed a certain level of knowledge and respect. Curiously, our findings revealed participants were much more interested in being honest and proving their worth.

We also acknowledge the anthropology of technology and concepts of the biotechnical embrace.^12^ The overconfidence in modern technology, like recording tools to improve services, should be countered by more fully considering use and value before implementation. As demand for richer patient engagement increases, so should the need to translate patient context into practice.^20^ Social asymmetries between clinicians and patients^21^, ^22^ prevent transparent communication.^23^ We acknowledge foundational work in sociology and social psychology on the effects of social distance on patient‐provider communication^24^ as well as more recent contributions debating how these impact different groups.^25^ A review of efforts to improve patient engagement suggest that many are tokenistic.^26^ Short decision support tools provided to patients at the point of care have been shown to work as “flexible artefacts” to improve patient‐centred communication.^10^ These studies argue that although wide social and cultural distances influence social interactions and clinical decisions, interventions that change usual clinical patterns of communication are helpful at bridging these gaps. While recordings may not “empower” marginalized individuals, recordings seen as “artefacts” may help validate their lived experiences enough to disrupt clinical misperceptions.

### 5.1 Practice implications

The clinical experience of individuals living on the margin is often shaped by assumptions. Evidence that power imbalances associated with patient‐provider relationships can be used to heal^27^ and humiliate^28^ circumscribes the responsibility of clinicians. Our data highlight how marginalized patients want doctors to recognize their hardship and believe they are good and honest. This interest may be obscured by the social distance experienced by patients, who may feel intimidated by obscure “doctor talk” or reluctant to ask questions for fear of being seen as “difficult.” Open recording with a smartphone may provide a potent tangible and symbolic reminder to clinicians that marginalized patients want health‐related information. With a recording that holds both patients and doctors accountable, the burden of proof can be shared.

## Conflict of Interest

The authors declared that they have no financial or other conflict of interests.

## Appendix 1 Empowering Rural Patients in Health‐Care Encounters

### Sample Interview Topic Guide

Thank you so much for agreeing to participate in this interview. We are interested in learning about your perspectives on communicating with health‐care providers and your perspectives on strategies to improve interactions with health‐care providers. We are interested in learning about these issues from your point of view; there are no right or wrong answers.

To begin, I will describe something that some patients are beginning to do during their appointments with health‐care providers. We are interested in getting your reactions to, and thoughts about, this: Some patients are using their smartphones to audio‐record their interactions with health‐care providers.

What do you think about this behaviour? What is your reaction to this idea?

Why do you think some patients are doing this? What might be the harms or benefits of this behaviour?

In some cases, patients are doing this covertly and without permission. But, we could imagine that audio recording clinical encounters could be done openly and with the health‐care provider's knowledge and permission.

What do you think about this? What is your reaction to the idea of openly, and with permission, recording clinical encounters?

Is this something that you would ever consider doing? Why/why not?

How do you think your health‐care providers would react? Explain.

There are a number of reasons why people might want a recording of their health‐care appointment. For example, people might have trouble remembering medication instructions or be confused by medical jargon. Being able to listen to a recording of the health‐care visit could be helpful to confirm treatment instructions or to clarify medical terminology. Does this change your view of audio recording health‐care encounters? In what ways?

Thinking of your own experiences, how might audio recording clinical encounters be useful to you? Could you think of any reasons this might not be useful or even difficult for you?

Would you be willing to try openly, and with permission, recording a visit with a health‐care provider? Why/why not?
